# Computational Modeling Reveals Optimal Strategy for Kinase Transport by Microtubules to Nerve Terminals

**DOI:** 10.1371/journal.pone.0092437

**Published:** 2014-04-01

**Authors:** Yen Ling Koon, Cheng Gee Koh, Keng-Hwee Chiam

**Affiliations:** 1 Mechanobiology Institute, National University of Singapore, Singapore, Singapore; 2 Interdisciplinary Graduate School, Nanyang Technological University, Singapore, Singapore; 3 School of Biological Sciences, Nanyang Technological University, Singapore, Singapore; 4 A*STAR Bioinformatics Institute, Singapore, Singapore; Stanford University School of Medicine, United States of America

## Abstract

Intracellular transport of proteins by motors along cytoskeletal filaments is crucial to the proper functioning of many eukaryotic cells. Since most proteins are synthesized at the cell body, mechanisms are required to deliver them to the growing periphery. In this article, we use computational modeling to study the strategies of protein transport in the context of JNK (c-JUN NH2-terminal kinase) transport along microtubules to the terminals of neuronal cells. One such strategy for protein transport is for the proteins of the JNK signaling cascade to bind to scaffolds, and to have the whole protein-scaffold cargo transported by kinesin motors along microtubules. We show how this strategy outperforms protein transport by diffusion alone, using metrics such as signaling rate and signal amplification. We find that there exists a range of scaffold concentrations for which JNK transport is optimal. Increase in scaffold concentration increases signaling rate and signal amplification but an excess of scaffolds results in the dilution of reactants. Similarly, there exists a range of kinesin motor speeds for which JNK transport is optimal. Signaling rate and signal amplification increases with kinesin motor speed until the speed of motor translocation becomes faster than kinase/scaffold-motor binding. Finally, we suggest experiments that can be performed to validate whether, in physiological conditions, neuronal cells do indeed adopt such an optimal strategy. Understanding cytoskeletal-assisted protein transport is crucial since axonal and cell body accumulation of organelles and proteins is a histological feature in many human neurodegenerative diseases. In this paper, we have shown that axonal transport performance changes with altered transport component concentrations and transport speeds wherein these aspects can be modulated to improve axonal efficiency and prevent or slowdown axonal deterioration.

## Introduction

Computational modeling of the dynamics of intracellular signaling pathways is an area of active research. Chemical equations such as the law of mass action or other higher-order reactions have been used to simulate the various intermolecular interactions involved in signaling pathways [1], [2]. Such equations can be solved analytically or numerically, and their steady state values can be further analyzed to gain deeper insights into the functions of the signaling pathways. Unfortunately, such analysis, when performed with the inherent assumption of the cell as a homogeneous mixture or as a well-stirred reactor, neglect the heterogeneous environment within a cell. The importance of such heterogeneity has been increasingly exemplified by evidence supporting the spatial localization of signaling proteins in a cell as an important contributor to the cell's proper functioning [3], [4]. To this end, models have been extended to include compartmentalization to account for interactions happening in non-interacting compartments such as the membrane, cytoplasm, and nucleus [5], [6]. Other models added diffusion to their reaction equations to include for molecular diffusion [7], [8]. Yet other models account for more specific forms of spatial variation such as subdiffusion to mimic the motion of proteins in a dense and crowded cytosol [9]–[11]. However, an aspect of signaling that contributes to spatial variation has to date not been well studied: the assisted transport of signaling proteins by cytoskeletal-associated motor proteins. Even though, computational studies concerning motor proteins in transport have been investigated with regards to vesicle transport [12] and with respect to heterogeneity matter distribution [13], [14], studies exploring the interplay between cytoskeletal transport and signaling is lacking. This manner of transport and the significance it plays in signaling will be the focal point of this article.

Such transport of proteins and organelles is especially important in neuronal cells. Most axonal proteins are synthesized within the neuronal cell body and mechanisms need to be in place to direct these proteins to the growing axon tips [15]. The complexity of transport is magnified by the sheer length of the distance involved in axonal transport. Axons of sciatic nerve cells have been reported to achieve lengths of more than one meter. Studies examining the molecular components of axonal transport have uncovered two classes of motor proteins that exist to transport cargo proteins along the cytoskeleton. Kinesin mainly governs anterograde axonal transport and transport mitochondria, transport vesicles and synaptic precursors from the cell body towards the synapse [16], [17]. On the other hand, dynein regulates retrograde axonal transport by carrying used components from the neurite tips back to the cell body for degradation and recycling [18]. These proteins govern two different modes of transport, namely, fast axonal transport and slow axonal transport. Membrane-spanning proteins or proteins possessing anchoring domains are packaged into vesicles and transported via fast axonal transport achieving rates of 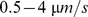. Slow axonal transport moves cytoskeletal and cytosolic proteins at average rates of 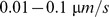 [19]–[21].

Often, proteins that are transported by motor proteins are also bound to scaffold proteins. Scaffold proteins have been known to interact and/or bind with various players of a signaling pathway and to tether them into complexes. In doing so, they regulate signal transduction and aid in localization of signaling cascades to specific parts of the cell. Signal activation by irrelevant stimuli can also be prevented, thus providing the cell with spatial and temporal control of signaling [22]. Computational models have shown that scaffold proteins are capable of amplifying signals for a limited range of scaffold protein concentrations [23], [24]. The biphasic dependence of signaling activity on the concentration of the scaffold protein has been verified experimentally for the prototypical scaffold protein, Ste5, in yeast cells [25]. However, it is unclear if such biphasic behavior of scaffold proteins exists in the presence of cytoskeletal transport.

One specific example of a signaling cascade that makes use of both scaffold proteins and motor proteins is the JNK (c-JUN NH2-terminal kinase) signaling pathway. The JNK group of mitogen-activated protein (MAP) kinases modulate a number of cellular processes in mammalian cells such as early embryonic development, apoptosis, oncogenic transformation and the immune response [26] and can be activated by environmental stress or inflammatory cytokines [27]. The JNK signaling module consists of various components including the mixed-lineage kinase (MLK) groups of MAP kinase kinase kinases (MAPKKKs), MAP kinase kinases (MAPKKs) like MAP kinase kinase 4 (MKK4) and MAP kinase kinase 7 (MKK7), and the MAP kinase, JNK. The JIP (JNK-interacting protein) group of scaffold proteins facilitate the signal transduction of the JNK signaling cascade by interacting with components of the JNK signaling pathway (including MLK, MKK7, and JNK) [28]–[30]. The JIP proteins have been demonstrated to be differentially located within cells. It accumulates in the growth cones at the tips of extended neurites [31]–[33] as well as within cell surface projections of cultured cells [30]. Prominent localization of JIP1 in synapses has been identified via immunocytochemical analysis of the brain [34]. Specific localization of the JNK signaling cascade to the cell periphery appears to play a crucial role in its function since subcellular organization of JIP1 is altered following stress exposure and disruption of the *Jip1* gene in mice prevented JNK activation [31]. Local activation of JNK primarily within axons is also induced during nerve injury. Activated JNK and adaptor protein Sunday Driver (syd, also known as JIP3) are then transported retrogradely, bringing about the idea that a mobile axonal JNK-syd complex may generate a transport-dependent axonal damage surveillance system [35].

JIP localization to the cell periphery could be modulated via its association to kinesin. In fact, JIP1, JIP2 and JIP3 have been identified as binding partners to kinesin using yeast two-hybrid procedure with kinesin light chain as bait [36]. Constructs of kinesin-1 or KIF5 that inhibit neurite tip localization of JIP also inhibit localization of MAPKKK scaffolded by JIP [36]. These support the notion that the JIP scaffold is preloaded with its kinase cascade prior to reaching its final destination of transport, differing from the conventional view that signaling molecules assemble on scaffolds at their final destination. The findings also reinforce the idea that signaling scaffolds, in addition to juxtaposing kinases in a cascade, are capable of carrying information about the trafficking and localization of the cascade [37]. Many kinesin superfamily proteins (KIFs) have been reported to reach speeds ranging from 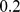 to 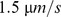, which are consistent with the speed of fast axonal transport *in vivo* [16], [19].

In this article, using the JNK signaling pathway as a model system, we study how the activity of JNK is being modified by being scaffolded via JIP1 and, in addition, transported to a distant part of the cell along the cytoskeleton via KIF5. Therefore, we seek to understand how the combined effects of both scaffolding and cytoskeletal transport modify signaling activity compared to the case if JNK is to diffuse to the distant part of the cell without scaffolding or cytoskeletal transport. We model the activation of JNK that is being scaffolded by JIP1 and then transported along the cytoskeleton via KIF5 by a set of reaction-diffusion-advection equations, and investigate how signaling rate and signal amplification are modified by the presence of scaffold and motor proteins. In Section II, we describe our model as well as the algorithm used to simulate the scheme effectively. Results of the simulations will be presented in Section III and discussed in Section IV. Finally, in Section V, we present our conclusions.

## Materials and Methods

**Figure 1:**
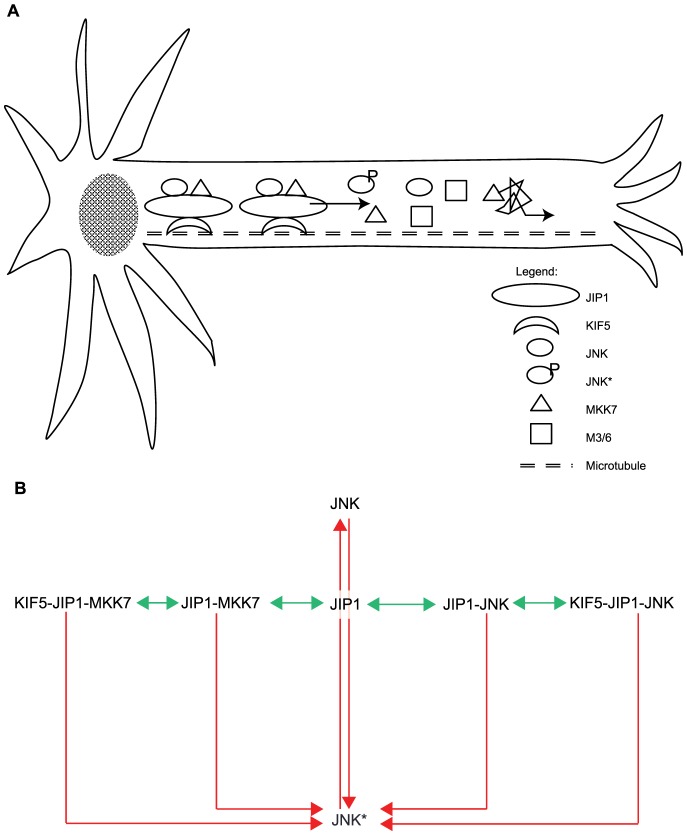
Schematic of model. (a) Schematic of a cell showing assisted-transport of proteins involved in the JNK signaling cascade, namely JNK and MKK7, by KIF5 (motor) via association with JIP1 (scaffold) from the cell body towards the cell periphery such as neurite tips. KIF5-bound proteins are transported along the microtubule track as depicted by the black arrow, indicating concerted direction of movement towards neurite tips. Proteins not bound to KIF5 diffuse as illustrated by the jagged black arrow. (b) Reactions modeled in the JNK signaling cascade. JIP1 serves as the scaffold for the recruitment of JNK and MKK7. It can be transported along microtubule tracks by the motor KIF5. Red arrows denote reactions with Michaelis-Menten kinetics. Green arrows denote reactions modeled using mass action kinetics.

In our model, the signaling protein JNK exists in either an inactive (unphosphorylated) or active (phosphorylated) state, denoted by JNK and JNK*, respectively. The activation of JNK is catalyzed by an upstream activated kinase MKK7 and the inactivation from JNK* to JNK is catalyzed by a phosphatase M3/6. Both the activation enzyme MKK7 and the inactive signaling protein JNK can bind to the scaffold protein JIP1. The scaffold protein JIP1 is assumed to possess catalytic properties such that the rate of activation of JNK by MKK7 is higher within the scaffold than that outside of the scaffold. Enhancement of catalysis within the scaffold has been observed experimentally where the prototypical scaffold Ste5 unlocks the Fus3 MAP kinase for activation by Ste7 MAPKK, thereby increasing the phosphorylation rate [38]. The scaffold protein, bare or complexed with either MKK7 or JNK or both, can bind to the motor protein KIF5. The motor protein and its cargo, i.e., the kinase-scaffold complex, are then transported through the cytosol along the microtubule cytoskeleton. Proteins that are not bound to the motor protein traverse the cytosol by diffusion, with a diffusion coefficient that is inversely proportional to the square root of their relative masses. The various molecular species JNK, JNK*, MKK7, M3/6, JIP1, and KIF5 and their interactions are depicted in Figure 1 with Table 1 containing the list of reactions and their rate constants.

**Table 1:** List of reactions and their corresponding rate constants.

Reactions	Rate Constants
JNK+MKK7 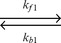 JNK-MKK7 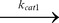 JNK*+MKK7	*k_f_*_1_ = 1.0/µM s†
	*k_b_*_1_ = 1.0/s†
	*k_cat_*_1_ = 0.1/s†
JIP1-JNK+MKK7 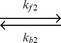 JIP1-JNK-MKK7 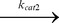 JIP1+JNK*+MKK7	*k_f_*_2_ = 1.0/µM s†
	*k_b_*_2_ = 1.0/s†
	*k_cat_*_2_ = 0.4/s†
JIP1-MKK7+JNK 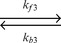 JIP1-JNK-MKK7 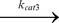 JIP1+JNK*+MKK7	*k_f_*_3_ = 1.0/µM s†
	*k_b_*_3_ = 1.0/s†
	*k_cat_*_3_ = *k_cat_*_2_ = 0.4/s†
JNK*+M3/6 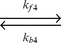 JNK*-M3/6 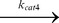 JNK*+M3/6	*k_f_*_4_ = 1.0/µM s†
	*k_b_*_4_ = 1.0/s†
	*k_cat_*_4_ = 0.1/s†
KIF5-JIP1-JNK+MKK7 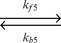 KIF5-JIP1-JNK-MKK7	*k_f_*_5_ = 1.0/µM s†
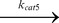 KIF5-JIP1+JNK*+MKK7	*k_b_*_5_ = 1.0/s†
	*k_cat_*_5_ = 0.4/s†
KIF5-JIP1-MKK7+JNK 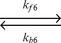 KIF5-JIP1-JNK-MKK7	*k_f_*_6_ = 1.0/µM s†
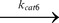 KIF5-JIP1+JNK*+MKK7	*k_b_*_6_ = 1.0/s†
	*k_cat_*_6_ = *k_cat_*_5_ = 0.4/s†
JNK+JIP1 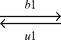 JIP1-JNK	*b*1 = 0.1/µM s†
	*u*1 = 0.1/s†
MKK7+JIP1 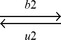 JIP1-MKK7	*b*2 = 0.1/µM s†
	*u*2 = 0.1/s†
JNK+KIF5-JIP1 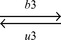 KIF5-JIP1-MKK7	*b*3 = 0.5/µM s†
	*u*3 = 0.1/s†
MKK7+KIF5-JIP1 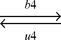 KIF5-JIP1-MKK7	*b*4 = 0.5/µM s†
	*u*4 = 0.1/s†
KIF5+JIP1 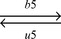 KIF5-JIP1	*b*5 = 0.5/µM s‡
	*u*5 = 0.1/s‡
KIF5+JIP1-JNK 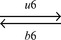 KIF5-JIP1-JNK	*b*6 = 0.5/µM s‡
	*u*6 = 0.1/s‡
KIF5+JIP1-MKK7 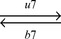 KIF5-JIP1-MKK7	*b*7 = 0.5/µM s‡
	*u*7 = 0.1/s‡

† Values of rate constants were chosen to be similar to estimates in [23].

‡ Values of rate constants were estimated.

In the JNK signaling cascade, three kinases are successively activated under stimulus. The cascade starts with activation of MAPKKKs such as MLK3 which go to on to phosphorylate and activate the MAPKKs MKK4 and MKK7 which finally phosphorylate and activate JNK [26], [39]. However, in our model, we only consider the final two kinases in the cascade, i.e., the activation of JNK by activated MKK7. By focusing on the last step of activation in the signaling pathway, we believe that the complicated reaction dynamics involved in activating JNK can be abridged, thus providing a clearer analysis regarding the behavior of the JNK signaling cascade making use of scaffold proteins for recruitment and motor proteins for transport. Association of M3/6 with JIP1 has also been neglected since only a small proportion of JIP1 is complexed with M3/6 in resting neuronal cells [40]. In the model, we also assumed that binding of JIP1 to KIF5 is sufficient for activation of motor even though both JIP1 and fasciculation and elongation protein 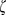
1 (FEZ1) are necessary for KIF5 activity [41]. Furthermore, we are concerned with the delivery of JNK to the cell periphery and thus neglect reactions involving JNK at the nerve terminals.

In the model, the reactions in Table 1 occur in a radial slice of the cell, i.e., a one-dimensional domain, 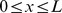, with the cell centre and cell periphery located at 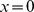 and 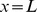, respectively. This one dimensional space is then discretized into discrete mesh elements each of size 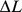 where the set of reactions in Table 1 take place at each discrete mesh element. In this article, we have used a discrete mesh of 200 elements where 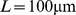 (and therefore 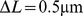).

Species that are not bound to KIF5 move by diffusion only. They follow the Neumann boundary condition 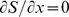 at 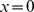 and 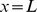. Species transported along the cytoskeleton, namely those that are motor protein-associated, follow the Dirichlet boundary condition 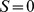 at 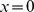. The motor protein KIF5, when not bound to cargo, is assumed to be immobile since KIF5 is present in a folded conformation that results in autoinhibition of the N-terminal motor domain by C-terminal tail domains in the absence of cargo [42], [43].

The initial distribution of all the species with the exception of M3/6 is assumed to follow a Gaussian distribution centered at 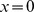. We used a standard deviation of 0.158 µm for KIF5 and 0.5 µm for the other species. On the other hand, M3/6 is assumed to be homogeneously dispersed throughout the domain with a uniform concentration of 1.0 µM. The initial distribution of proteins is listed in Table 2. Diffusion coefficients of the species are also listed in Table 2.

**Table 2:** Diffusion coefficients and initial distribution of all species modeled.

Molecular Species	Initial Distribution (µM)	Diffusion Coefficient Notation	Diffusion Coefficient⊕ (µm^2^/s)
JNK	[JNK] (*x*) = 10 exp(−*x* ^2^/2(0.5)^2^)	D_JNK_	10
JIP1	[JIP1] (*x*) = (0 *to* 20) exp (−*x* ^2^/2(0.5)^2^)	D_JIP1_	10
MKK7	[MKK7] (*x*) = 1.6 exp(−*x* ^2^/2(0.5)^2^)	D_MKK7_	10
JNK*	[JNK*] (*x*) = 0	D_JNK*_	10
M3/6	[M3/6] (*x*) = 0.1	D_M3/6_	10
JIP1-JNK	[JIP1-JNK] (*x*) = 0	D_JIP1–JNK_	10
JNK-MKK7	[JNK-MKK7] (*x*) = 0	D_JNK–MKK7_	7.07
JIP1-MKK7	[JIP1-MKK7] (*x*) = 0	D_JIP1–MKK7_	10
JIP1-JNK-MKK7	[JIP1-JNK-MKK7] (*x*) = 0	D_JIP1–JNK–MKK7_	5.77
JNK*-M3/6	[JNK*-M3/6] (*x*) = 0	D_JNK*–M3/6_	10
KIF5	[KIF5] (*x*) = 10 exp(−*x* ^2^/2(0.16)^2^)	-	-
KIF5-JIP1	[KIF5-JIP1] (*x*) = 0	-	-
KIF5-JIP1-JNK	[KIF5-JIP1-JNK] (*x*) = 0	-	-
KIF5-JIP1-MKK7	[KIF5-JIP1-MKK7] (*x*) = 0	-	-
KIF5-JIP1-JNK-MKK7	[KIF5-JIP1-JNK-MKK7] (*x*) = 0	-	-

⊕ Diffusion coefficients were chosen to be similar to estimates in [72].

We solve the set of reaction-diffusion-advection equations listed in Table 3 numerically using the Forward-Time Central-Space (FTCS) scheme for the diffusion equations and the second order Lax-Wendroff scheme for the advection equations. From our simulations, we answer the following questions. First, how does the activated kinase JNK* accumulate at the periphery of the cell, 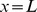, from a source of inactivated kinase JNK initially clustered at the centre of the cell, 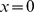. Two possible mechanisms could happen: the inactive kinase JNK diffuses around the cytosol, depending on chance encounters with the upstream kinase MKK7 to become activated. The activated kinase JNK* then also diffuses until it reaches its destination. The aforementioned mechanism would rely entirely on diffusion without any dependence on motor proteins. In the second mechanism, cytoskeletal transport via motor proteins are involved. The inactive kinase JNK, while undergoing diffusion around the cytosol, chances upon and associates with the scaffold JIP1. In some cases, the scaffold will already have associated with MKK7, and so, the kinase complexed with these scaffolds will be activated. The scaffold complex diffuses and can encounter and bind to the motor protein KIF5. The whole motor protein and cargo complex is then transported along the microtubule cytoskeleton to their destination.

**Table 3:** Differential equations of all species modeled.

Molecular Species	Differential Equations◊
JNK	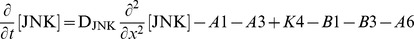
JIP1	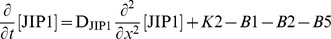
MKK7	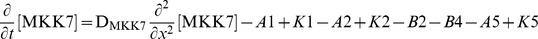
JNK*	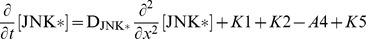
M3/6	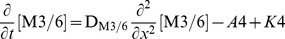
JIP1-JNK	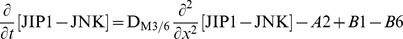
JNK-MKK7	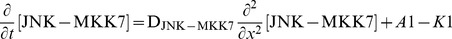
JIP1-MKK7	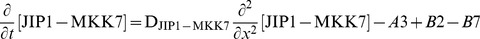
JIP1-JNK-MKK7	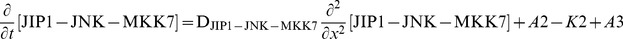
JNK*-M3/6	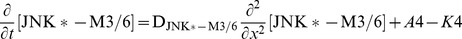
KIF5	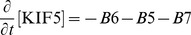
KIF5-JIP1	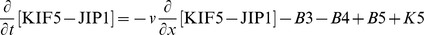
KIF5-JIP1-JNK	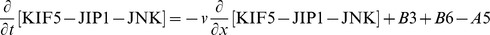
KIF5-JIP1-MKK7	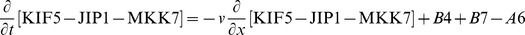
KIF5-JIP1-JNK-MKK7	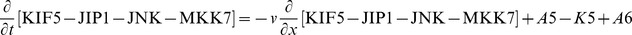

◊ Notations in the table are represented by the following:

*A*1 = *k_f_*_1_⋅[JNK]⋅[MKK7] −*k_b_*_1_⋅[JNK-MKK7].

*A*2 = *k_f_*_2_⋅ [JIP1-JNK]⋅[MKK7] −*k_b_*_2_⋅[JIP1-JNK-MKK7].

*A*3 = *k_f_*_3_⋅[JIP1-MKK7]⋅[JNK] −*k_b_*_3_⋅[JIP1-JNK-MKK7].

*A*4 = *k_f_*_4_⋅[JNK*]⋅[M3/6] −*k_b_*_4_⋅[JNK*-M3/6].

*A*5 = *k_f_*_5_⋅[KIF5-JIP1-JNK]⋅[MKK7] −*k_b_*_5_⋅[KIF5-JIP1-JNK-MKK7].

*A*6 = *k_f_*_6_⋅[KIF5-JIP1-MKK7]⋅[JNK] −*k_b_*_6_⋅[KIF5-JIP1-JNK-MKK7].

*K*1 = *k_cat_*_1_⋅[JNK-MKK7].

*K*2 = *k_cat_*_2_⋅[JIP1-JNK-MKK7].

*K*3 = *k_cat_*_3_⋅[JIP1-JNK-MKK7].

*K*4 = *k_cat_*_4_⋅[JNK*-M3/6].

*K*5 = *k_cat_*_5_⋅[KIF5-JIP1-JNK-MKK7].

*B*1 = *b*1⋅[JNK]⋅[JIP1] −*u*1⋅[JIP1-JNK].

*B*2 = *b*2⋅[MKK7]⋅[JIP1] −*u*2⋅[JIP1-MKK7].

*B*3 = *b*3⋅[KIF5-JIP1]⋅[JNK] −*u*3⋅[KIF5-JIP1-JNK].

*B*4 = *b*4⋅[KIF5-JIP1]⋅[MKK7] −*u*4⋅[KIF5-JIP1-MKK7].

*B*5 = *b*5⋅[JIP1]⋅[KIF5] −*u*5⋅[KIF5-JIP1].

*B*6 = *b*6⋅[JIP1-JNK]⋅[KIF5] −*u*6⋅[KIF5-JIP1-JNK].

*B*7 = *b*7⋅[JIP1-MKK7]⋅[KIF5] −*u*7⋅[KIF5-JIP1-MKK7].

Next, we want to understand the relative importance between protein diffusion and cytoskeletal transport in the arrival and accumulation of JNK* at the cell periphery. Specifically, we are interested in how two parameters, the concentration of scaffold protein JIP1 and the speed 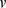 of motor protein KIF5, modify signaling activity. These two parameters can be expressed in dimensionless forms, 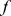 and 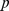 respectively, where 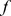 is the ratio of the initial concentration of JIP1 to the initial concentration of JNK,
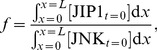
(1)and 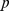 the ratio of the rate of advection to the rate of diffusion,
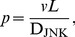
(2)where 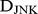 is the diffusion coefficient of JNK. We can also view 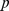 as the inverse ratio of the time of transport by motor proteins to the time of transport by diffusion to the same distance.

## Results

### Scaffolded cytoskeletal transport can result in a higher JNK* activation than diffusion

**Figure 2:**
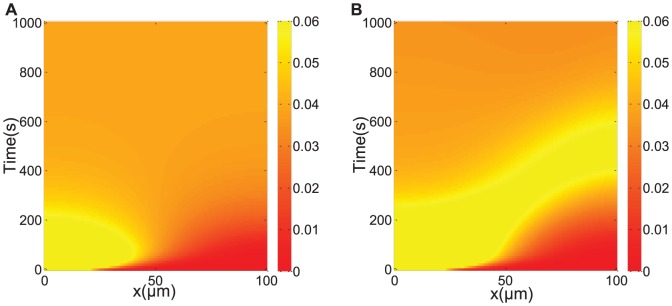
Kymograph of JNK* activity. Kymograph plots of JNK* activity (red = low, yellow/white = high) for (a) 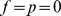 and (b) 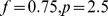. Comparison between (a) and (b) reveals that JNK that is scaffolded and transported on the cytoskeleton (case (b)) can result in delivery of JNK and activation to JNK* at the cell periphery more efficiently that relying on diffusion alone (case (a)). Maximum value of JNK* attained at the cell periphery in (b) is 0.0645 µM which is more than that achieved by diffusion alone (0.0389 µM). Also, in (b), JNK* at the cell periphery attains its maximum value at 490 seconds whereas diffusion alone requires 3300 seconds.

We first solve for the purely diffusive scenario where 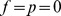, i.e., no scaffolds are present and no motor proteins are present (or more accurately, motor proteins are present but are stationary), and transport takes place by diffusion only. We next compare this control scenario to the scenario when both 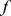 and 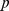 are not equal to 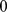, i.e., when there is scaffolding and transport by the motor proteins on the cytoskeleton. Space-time kymographs of the level of JNK* concentration are shown in Figure 2(a) and (b) for two scenarios, respectively. In panel (a), as time progresses, JNK* moves about purely by diffusion. Compare this to the scenario in panel (b), where there are both associations to JIP1 and cytoskeletal transport by KIF5. Thus, JNK* activity moves at a constant speed towards the cell periphery. Maximum value of JNK* attained at the cell periphery is 0.0645 µM which is more than that achieved by diffusion alone (0.0389 µM). At maximum signaling activity at the cell periphery, JNK* is also observed to be localized to the periphery for motor proteins-assisted transport whereas JNK* is spread across the entire cell length for the purely diffusive case. This is supported by [36] where localization of dual leucine zipper kinase (DLK), a member of the MLK family of kinases, is abolished when kinesin is inhibited. Furthermore, when kinesin is not inhibited, a higher concentration of DLK is observed at the neurite tip compared to the case when kinesin is inhibited [36]. Furthermore, in panel (b), JNK* attained its maximum value after 490 seconds whereas in panel (a), JNK* requires a far longer time of 3300 seconds to reach maximum value. These results suggest that scaffolded cytoskeletal transport can indeed result in a higher level of JNK* activation at the cell periphery than diffusion alone. This is supported in [44] where the combined effect of small protein diffusion coefficients and rapid dephosphorylation leads to hampering of information transfer and it is suggested that assembling protein kinases on a scaffold and using motor proteins to transport these signaling complexes can lead to a more efficient way of delivery.

We shall now proceed to quantify the transport activities more carefully. In particular, we define and make use of two metrics, namely, signaling rate and signal amplification. Signaling rate, 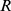 is defined to be the inverse of the time needed for the JNK* to reach its maximum concentration at the cell periphery,
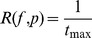
(3)where 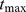 is the time at which maximum signaling activity is achieved at the cell periphery, 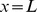. Next, signal amplification, 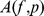, is defined to be the ratio of the maximum concentration of JNK* achieved at the cell periphery over time for a particular value of 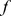 and 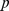 to the maximum concentration of JNK* when there are no scaffold and motor proteins present, also at the cell periphery. Signal amplification measures the extent to which signaling activity is enhanced by the combined effect of scaffolding and cytoskeletal transport,
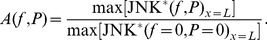
(4)


### Increase in speed of cytoskeletal transport does not always lead to an increase in signaling rate

**Figure 3:**
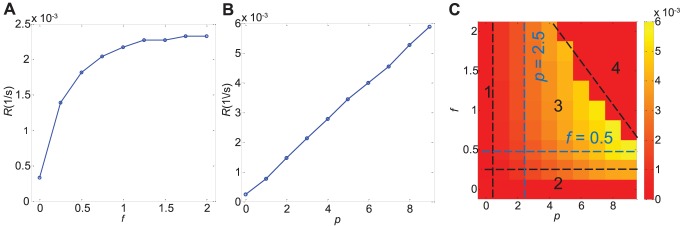
Signaling rate for various values of 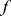 and 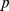. (a) Signaling rate, 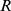, for fixed 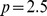 increases with increasing 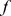. (b) Signaling rate for fixed 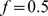 increases with increasing 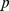. (c) Signaling rate for a range of 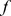 and 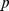. Four distinct regions can be distinguished (labeled 1 to 4) and demarcated by black dashed lines. Region 1 is defined by 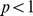 and Region 2 by 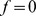. Signaling rate is low in Regions 1 and 2. In moderate values of 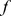 and 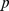 lies Region 3 where signaling rate is high and increases with both 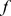 and 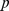. Region 4 lies beyond Region 3 and is characterized by low signaling rate even at high values of 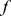 and 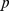. The blue dashed lines denote the cases illustrated in (a) and (b).

If we fix 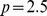 and vary 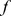, we see that the signaling rate increases as JIP1 concentration or 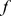 increases; see Figure 3(a). KIF5 motors are capable of motion only when it is associated with JIP1. When JIP1 concentration increases, more KIF5 motors are activated. An increase in activated KIF5 will lead to the delivery of more associated kinases to the cell periphery, leading to an increase in signaling rate.

Similarly, if we now fix 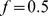 and vary 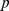, we see that signaling rate increases with 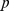. An increase in 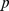 will lead to an increase in the transport of any JNK or MKK7 bound to KIF5 via JIP1. Kinases can be delivered to the cell periphery at a shorter time at larger 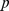 values leading to improved signaling rate.

Intuitively, one would expect that an increase in the motor speed (or equivalently, 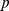) will always result in an improvement in the signaling rate. However, as we show in Figure 3(c), such is not the case. The signaling rate is not observed to be monotonically increasing with 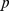 but instead dependent on both 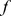 and 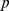. In fact, we can identify four distinct regions as denoted in Figure 3(c):

1. When 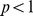 (meaning transport by motor proteins is slower than transport by diffusion, and denoted by Region 1), the signaling rate is low regardless of the value of 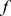 or scaffold concentration. This is attributed to the slow movement of KIF5. Slow movement of KIF5 will lead to the slow delivery of associated kinases to the cell periphery causing the signaling rate to be low.
2. When 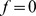 (no JIP1 scaffold proteins, and denoted by Region 2), JNK and MKK7 are not transported by KIF5 in the absence of JIP1 since JIP1 scaffolds are required as a linker to bind JNK and MKK7 to KIF5. Thus, transport of JNK and MKK7 to the cell periphery will depend only on diffusion, resulting in a low signaling rate.
3. For moderate values of 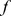 and 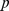 (denoted by Region 3), the signaling rate increases with increases in both 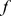 and 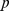. Due to the inability of KIF5 to move unless associated with cargo, increasing concentration of JIP1 will lead to an increase in cargoes capable of activating motion in KIF5, thus improving signaling rate. Increasing 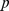 also improves signaling rate since KIF5 motors can transport its associated JNK and MKK7 at a faster speed to the cell periphery. The two cases of fixing 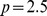 and varying 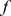 and fixing 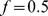 and varying 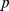 discussed above both lie within Region 3.
4. For high values of both 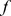 and 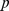 (denoted by Region 4), the signaling rate actually decreases to a low value. This can be explained as follows. When the cell contains a large amount of JIP1 scaffolds, most of them will predominantly be empty instead of being bound to JNK or MKK7. Cargoes loaded and transported by KIF5 would therefore be empty scaffolds. In such a situation, JNK and MKK7 will move via diffusion leading to poor signaling rate. At fast motor speed, KIF5 motors are moving too quickly for binding of JNK and MKK7 to take place. JNK and MKK7 will once again rely on diffusion to reach the cell periphery.

An interesting feature here is that the boundary demarcating high signaling rates (Region 3) from low signaling rates (Region 4) depends on both 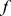 and 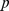. This would mean that for the cell to achieve high signaling rate at high speeds, low 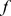 is required, and, vice versa, a low value of 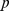 is needed to attain high signaling rate at high values of 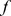. At low 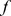, JIP1 would predominantly be in the form complexed with its kinases, either JNK, MKK7 or both. Thus, KIF5 will associate with JNK and MKK7 at low 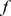 and can transport these kinases towards the cell periphery even at high 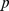. At high 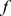, KIF5 would largely be associated with empty JIP1 without JNK and MKK7. In such a situation, if speed of cytoskeletal transport is faster than the speed of binding of JNK and MKK7 to KIF5-JIP1, JNK and MKK7 kinases would not be bound to KIF5 and have to rely on diffusion to reach the cell periphery. Thus in order to achieve high signaling rate at large 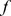, low 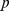 is necessary. In summary, Region 3 is the region where cytoskeletal transport is able to deliver kinases to the cell periphery and Region 4 is the region where cytoskeletal transport, though present, is ineffective in transporting kinases and kinases move to the cell periphery by diffusion. A sharp jump in signaling rate between Region 3 and Region 4 thus exists since speed of cytoskeletal transport is a lot faster than speed of diffusion. The boundary separating Regions 3 and 4 can be adjusted by modifying the strength of binding of free kinases with KIF5-JIP1. Indeed, the boundary between Region 3 and Region 4 is shifted upwards in the presence of stronger binding. (Data not shown.) Increasing binding strength of JNK and MKK7 to KIF5-JIP1 thus serves to increase association of kinases to KIF5 allowing for high signaling rates at fast cytoskeletal transport speed.

### An optimal scaffold protein concentration and optimal motor speed exist for which signal amplification is maximal

**Figure 4:**
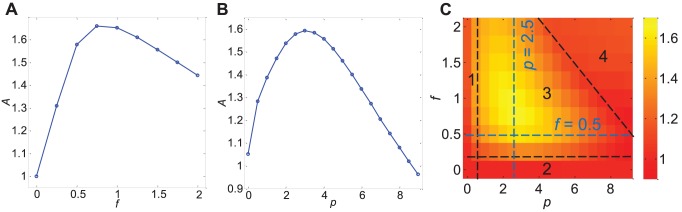
Signal amplification for various values of 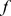 and 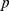. (a) Signal amplification, 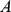, for fixed 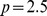 and varying 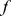. (b) Signal amplification for fixed 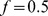 and varying 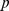. In both cases, there exists a maximum value of 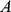 and hence an optimal value of 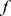 and 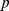 to attain this maximum. (c) Signal amplification for a range of 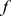 and 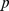. Highest value of signal amplification was attained at moderate levels of 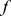 and 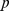. Four distinct regions can be identified (labeled 1 to 4) and demarcated by black dashed lines. Region 1 is defined by 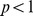 and Region 2 by 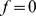. Signal amplification is low in Regions 1 and 2. In moderate values of 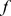 and 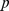 lie Region 3 where signal amplification is increased and exhibits a biphasic behaviour in both 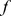 and 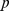. Region 4 lies beyond Region 3 and is characterized by low signal amplification even at high values of 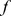 and 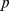. The blue dashed lines denotes the cases illustrate in (a) and (b).

Next, we look at the signal amplification, 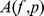, for different values of 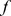 and 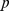. If we fix 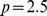 and vary 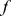, we see that the signal amplification 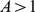 when 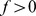; see Figure 4(a). As the concentration of JIP1 or 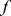 increases, signal amplification increases. However, there exists a maximum for signal amplification 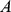 at 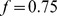. Beyond 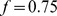, if the concentration of JIP1 is increased further, signal amplification decreases. In this case, continued increase in scaffolds result in dilution of kinases lowering signaling activity. Thus, there exists an optimal scaffold concentration where amplification of signaling activity is maximal. This observation, when there is cytoskeletal transport, 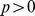, is consistent with the stationary case obtained by previous authors [23], [25]. We have now demonstrated that this result is still true even in the presence of cytoskeletal transport. Similar profiles are observed for other values of 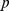 in Figure 4(b).

Similarly, if we now fix 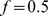 and vary 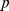, we see that there is signal amplification, 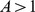, when 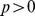. This amplification increases with increasing 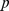; see Figure 4(b). At small values of 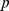, an increase in 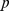 will lead to an increase in the transport of any JNK or MKK7 bound to KIF5 via JIP1. Bound kinases can be delivered to the cell periphery in a shorter time thus less time is available for dephosphorylation events which inactivate JNK*. A maximum value of signal amplification occurs when almost all the kinases are scaffold-bound and the corresponding complexes are attached to the motor proteins moving towards the cell periphery. As 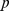 is increased further, signal amplification decreases. In this case, only a few JNK and MKK7 associated scaffold complexes will be actively transported since the speed of translocation of the motor proteins is faster than that of kinase/scaffold-motor binding. Thus, there exists an optimal cytoskeletal transport speed where amplification of signaling activity is maximal.

Similar profiles are observed for other values of 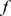 and 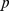 as observed in Figure 4(c). Likewise for the signaling rate plot in Figure 3(c), four distinct regions can be distinguished from Figure 4(c). Low amplification is observed in Region 1 and Region 2 defined by 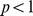 and 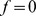, respectively. Signal amplification increases and displays a biphasic behavior with respect to 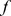 and 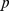 in Region 3 at moderate values of 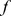 and 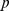. Lastly, Region 4 lies beyond Region 3 at high 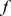 and 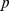. Signal amplification is low within Region 4. A smooth transition occurs between Region 3 and Region 4. At high 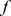 and 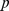 values, amount of kinases carried by KIF5 decreases with increase in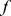 and 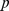. Consequently, amount of kinases that reaches the cell periphery by diffusion increases as 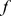 and 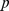 increases. Thus amplification changes gradually from Region 3 to Region 4 since the magnitude of kinase delivery by diffusion and cytoskeletal transport changes smoothly between the two regions.

### Optimum scaffold protein concentration and optimal cytoskeletal transport speed depend on signaling parameters

We seek to understand how the values of scaffold concentration 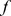 and motor speed 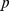 which gives optimal signaling rate and signal amplification depend on the state of the cell.

**Figure 5:**
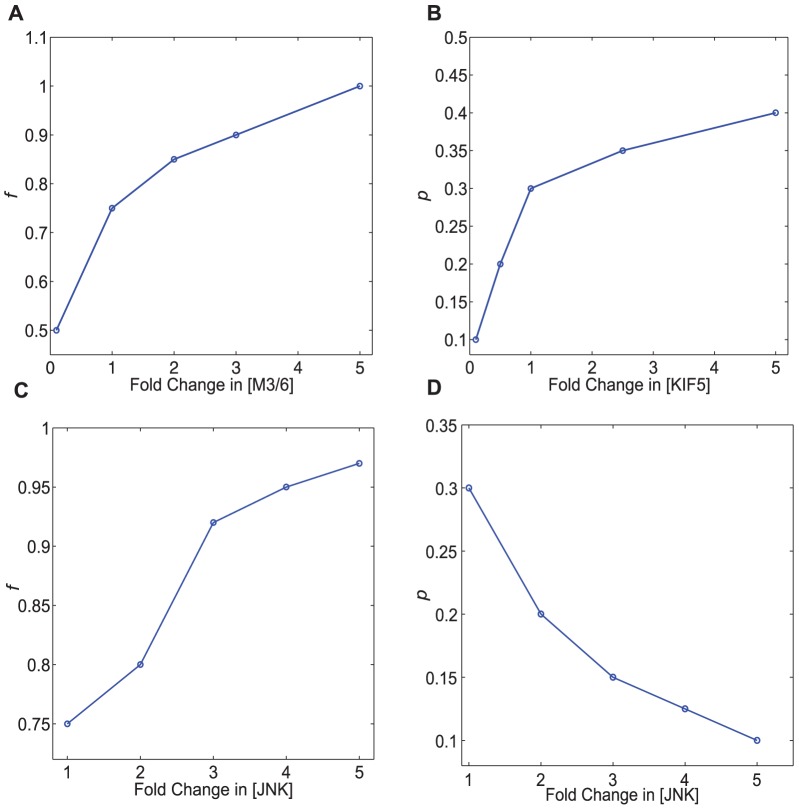
Values of 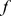 and 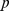 for which signal amplification is optimum depend on signaling parameters. The value of 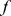 for which signal amplification is optimum increases with (a) increasing M3/6 concentration, and (c) increasing JNK concentration. The value of 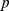 for which signal amplification is optimum increases with (b) increasing KIF5 concentration. (d) However, this value of 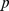 decreases with increasing JNK concentration.

Increasing concentration of M3/6 increases the value of 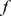 necessary for maximum signal amplification as shown in Figure 5(a). Signaling pathways are often inactivated by enzymes that reverse the activation state and/or induce the degradation of signaling components. Scaffolds have been proposed to prevent activated signaling molecules from inactivation and/or degradation. Mathematical modeling has shown that kinases in a cascade without scaffolds have a higher probability of being dephosphorylated by phosphatases before they are even able to phosphorylate downstream targets [45]. Therefore, in the presence of higher concentration of M3/6, more JIP1 scaffolds are needed to sequester JNK and MKK7 to increase the incidence of the forward reaction leading to a higher value of optimum 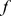 required for signal amplification.

A higher concentration of KIF5 motor protein increases the value of 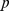 for maximum signal amplification as shown in Figure 5(b). Since KIF5 can only be activated when it is cargo-bound, when more KIF5 is present, more JIP1 and kinases complexed with JIP1 can bind to KIF5 prior to KIF5 movement along the cytoskeleton. Cytoskeletal transport can thus take place at a higher speed since more kinases are being bound to KIF5 at a higher concentration of KIF5.

Next, we look at how JNK concentration modifies the values of 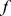 and 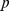 to yield optimal signaling. An increase in the amount of JNK implies that a higher concentration of scaffolds can be present before dilution of kinases occur leading to an increase in optimum 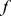 as seen in Figure 5(c). Unlike optimum 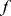, optimum 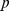 decreases as concentration of JNK increases as observed in Figure 5(d). At higher JNK concentration, cytoskeletal transport speed needs to be reduced to ensure that more JNK is bound onto KIF5 before KIF5 translocate along the cytoskeleton.

Thus, one can foresee a scenario where the cell upregulates JIP1 scaffolds and KIF5 motors when JNK concentration is increased at the cell body. Increasing the amount of JIP1 scaffolds serves to increase amplification of JNK* at the cell periphery while increasing KIF5 serves to increase the optimal speed of transport of associated kinases for faster delivery. JIP1 is observed to be upregulated with an increase in phosphorylation of JNK when GLUT1 (glucose transporter1) is overexpressed [46]. Genetic experiments performed in *C. elegans* also suggest that axonal transport depending on KIF5 is upregulated by the JNK pathway [17], [47]–[49]. Thus, it may be plausible that the JNK pathway may indeed upregulate both JIP1 and KIF5. On the other hand, there are reports that suggest that KIF5 can be phosphorylated by JNK which, upon phosphorylation, has a lower binding affinity to microtubules [50], [51]. This may be the root cause in spinal and bulbar muscular atrophy where JNK has been found to be abnormally activated leading to inhibition of fast axonal transport [50]. Thus, more work remains to be done to determine how the JNK pathway interacts with its binding partners such as JIP1 and KIF5.

## Discussion

The combination of scaffolding by JIP1 and transport by motor protein KIF5 can be summarized as follows. At low JIP1 scaffold concentration, few JNK are recruited to JIP1 for subsequent phosphorylation and transport by KIF5. Thus, majority of the JNK* reaches the axon terminals by free diffusion, resulting in low signaling rate and signal amplification. However, at high JIP1 concentrations, JNK and MKK7 are spread out too widely amongst the scaffold proteins, leading to ineffective phosphorylation and a corresponding suppression of phosphorylation activity in the entire system. Active transport of JNK* still occurs, although scaffold-assisted phosphorylation is now suppressed.

On the other hand, at low KIF5 speed, both forms of JNK (activated or unactivated) and MKK7 diffuse freely along the axons, such that they are far beyond encounter distance from KIF5 which are concentrated near the cell body. Under such circumstances, signaling proceeds via free diffusion coupled with limited active transport, resulting in low signaling rate and signal amplification. At high KIF5 speed, however, motor proteins translocate along the cytoskeleton before the kinases can bind onto the motor. Here, we witness the other extreme case whereby free diffusion coupled with limited active transport prevails.

**Figure 6:**
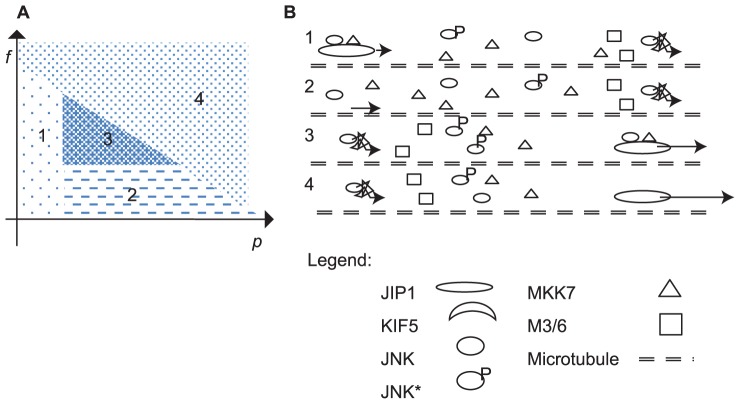
Phase diagram to summarize the possible strategies utilized by the cell. (a) In the first strategy denoted in Region 1 where 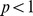, motor proteins are moving too slowly for efficient transport. Scaffold proteins are absent in the second strategy (Region 2, 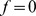) and thus proteins are unable to hitch onto motor proteins and have to rely on slow diffusion to reach the cell periphery. The optimum strategy is the third strategy denoted by Region 3 and involves moderate scaffold concentration and moderate motor speed. In the last strategy denoted by Region 4, proteins are not bound to motor proteins either because motor proteins are moving too quickly for binding to occur or scaffolds are in such abundance that binding between proteins and motors will preferentially be involving empty scaffolds.(b) Schematic illustrating how the proteins are moving in each of the four strategies.

Finally, an ideal scenario should comprise an optimum JIP1 concentration to concentrate both JNK and MKK7 effectively and an optimum KIF5 cytoskeletal transport speed, such that most of the corresponding scaffold complexes are recruited by the motor proteins and actively transported along the axons. Such a scenario is observed at the maxima region in the phase diagrams of signal amplification and lies within the high signaling rate region of the signaling rate plot. This is depicted in Region 3 of Figure 6(a) and Figure 6(b). Region 3 is characterized by moderate scaffold concentration and moderate motor transport speed. Highest JNK* signaling rate and largest JNK* signal amplification is contained within Region 3. In this region, the kinases are scaffold-bound and the corresponding complexes are attached to the motor proteins moving towards the cell periphery. In Region 1 defined by 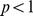, the kinases may be bound to scaffold but the speed of KIF5 is too slow for efficient transport. In Region 2, no scaffolds are present since 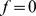. Transport of kinases to cell periphery relies on slow diffusion since the JIP1 scaffolds are absent to serve as linkers between kinases and KIF5. Region 4 is the region at high 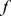 or high 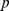. In this region, kinases are not bound to motors due to quenching of motors by excessive scaffolds for high 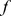 and insufficient time for kinase binding for high 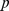. In both cases, kinases diffuse to the cell periphery instead of being transported along the cytoskeleton.

Even though our model is constructed specifically for the analysis of the JNK signaling cascade, we believe that our model is generic enough to be applied to other signaling pathways that also make use of scaffold proteins and cytoskeletal transport. Features extracted in our model such as the biphasic behavior in scaffold concentration and cytoskeletal transport speed should be universal features in other motor proteins-assisted scaffolded signaling complexes. In recent years, an increasing number of scaffold proteins that associate with motor proteins have been uncovered. A yeast-two-hybrid screen to identify proteins that interact with the KIF1C C-terminal domain identified proteins of the 14-3-3 family as binding partners [52]. The 14-3-3 family of proteins serves as scaffolds for a variety of signaling proteins such as phosphatases, kinases and transmembrane receptors. Costal2 (Cos2), a scaffold protein of the Hedgehog signal transduction pathway which recruits other signaling components, has also been reported to exhibit motility, thus functioning as a kinesin-like protein [53]. Cos2 is required for phosphorylation of Cubitus interruptus, Ci and Cos2 immunocomplexes contain protein kinase A (PKA), glycogen synthase kinase 3 (GSK3) and casein kinase I (CKI) [54]. Amyloid precursor protein (APP) has also been reported to bind to JIP proteins where the phosphorylation of APP by JNK was enhanced by the presence of the scaffold JIP in vitro and in cultured cells [55]–[57]. These findings support the notion that preassembled signal transduction cascades or transducisome are recruited to downstream motors in order to drive the regulated movement of attached cargo [58]–[61]. Thus, the model developed in this article can be used to study various signaling cascades and can potentially be used for in-depth analysis of other signaling complexes that remains to be discovered in the future.

The role of JIP1 in modulating the JNK pathway has been well studied. JIP1 was originally assumed to be an inhibitor of JNK. JIP1 has been shown to suppress signal transduction of the JNK pathway by competing with substrates that interact with JNK. JIP1 overexpression has also been proposed to be a cytoplasmic anchor for JNK as overexpression of JIP1 caused retention of JNK in the cytoplasm [62]. Recent discovery however reveals JIP1's role as a crucial scaffold protein for the MAP kinase cascade [28]. In this article, we have elucidated another role of JIP1 in regulating the dynamics of the JNK pathway. By binding both motor proteins and members of the JNK signaling cascade, JIP1 serves to enable cytoskeletal-assisted transport of JNK* allowing for greater signaling rate and signal amplification.

Understanding cytoskeletal-assisted protein transport is important, because axonal and cell body accumulation of organelles and proteins is a histological feature in many human neurodegenerative disease. Examples include polyQ aggregates in Huntington disease, synuclein in Lewy bodies found in Parkinson's disease, amyloid beta and tau protein deposits in Alzheimers disease. These observations suggest that defects in axonal transport may contribute to neuronal inclusions and plaques [63]. However, current research on neurodegenerative diseases is primarily focused on axonal transport defects, such as mutation of motor proteins, destabilization of microtubules, disruption of motor-cargo protein interactions and mitochondria dysfunction (leading to insufficient ATP supply for motor proteins). There has been little effort made to quantify axonal transport performance as a function of the intrinsic properties of the axonal transport machinery components. Previous studies exploring motor proteins in transport investigated its role in vesicle transport [12]. Motor proteins were found to improve the recycling of SNARE protein and to result in cell polarization. Advances regarding motor proteins were also made in terms of its contribution to density heterogeneity where it was found that the transport of motor protein can lead to a spontaneous distribution of matter and that these heterogeneities can be controlled via various factors such as the topology of the cytoskeletal network [13], [14]. In this article, we have shown that axonal transport performance changes with altered transport component concentrations and transport speeds. Such findings are important because it has been shown that differential activation time of JNK results in different induction of gene expression. Cell survival is promoted should JNK activation be early and transient. Prolonged JNK activation however leads to apoptosis [64]. Regulation of JNK temporal dynamics is thus critical to elicit an appropriate cellular response.

Finally, we discuss how the two parameters 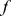 and 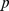 used in this article can be varied experimentally. To vary 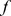 experimentally, JIP1 scaffolds can be up or down-regulated. While it is not easy to modify motor speed, we note that we only need to vary motor speed with respect to diffusion. Thus, an easier way to vary 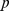 is to vary protein diffusivity by introducing dextran beads into the cytosol. Acetylation of microtubules could be another option to vary 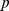 since it has been shown that hyper-acetylation of all microtubules in the central nervous system cell line Cath.a-differentiated (CAD) results in targeting of JIP1 to all neurite tips, nullifying the usual selectivity of its transport resulting in greater directed motion [65]. Tau protein implicated in Alzheimer's disease can also be introduced into the cell to inhibit kinesin transport since tau impedes anterograde transport [66]–[68]. This may be due to tau's effect on decreasing the attachment ability of kinesin to microtubules [69], [70] and/or decreasing the traveling distance of kinesin [71].

## Conclusion

We have studied computationally the various strategies that JNK may be transported from the cell body to the cell periphery. We have shown that binding to a scaffold JIP1 and then having the whole protein-scaffold cargo being transported by motor proteins KIF5 along the cytoskeleton is superior to relying on transport by protein diffusion, but only in a limited range of JIP1 concentration and KIF5 motor speed. We defined two metrics to quantify transport, namely signaling rate and signal amplification. It is only possible to achieve maximum amplification at a specific range of JIP1 concentration and KIF5 motor speed. These findings are summarized in Figure 6 which highlights the necessity of an optimum speed and scaffold concentration to achieve maximum signaling effectiveness.
